# Regulation of the plasminogen activator system in non-small cell lung cancer cell lines by growth factors EGF, TGF-alpha and TGF-beta.

**DOI:** 10.1038/bjc.1992.33

**Published:** 1992-02

**Authors:** H. H. Heidtmann, K. Havemann, R. Schwartz-Albiez

**Affiliations:** Department of Internal Medicine, Philipps University Marburg, Germany.

## Abstract

**Images:**


Br. J. Cancer (1992), 65, 154-156 © Macmillan Press Ltd., 1992

SHORT COMMUNICATION

### Regulation of the plasminogen activator system in non-small cell lung cancer cell lines by growth factors EGF, TGF-α and TGF-β

H.-H. Heidtmann[1], K. Havemann[1] & R. Schwartz-Albiez[2]

[1]*Department of Internal Medicine, Division of Hematology/Oncology, Philipps University Marburg, D-3550 Marburg;* [2]*German Cancer Research Center, D-6900 Heidelberg, Germany.*

Plasminogen activators (PA) have been postulated to play a role in tumour invasion due to their regulatory function in fibrinolysis and degradation of extracellular matrix components (Tanaka *et al.*, 1977; Cajot *et al.*, 1989). PA-mediated degradation is modulated by their specific inhibitors (Laiho & Keski-Oja, 1989), urokinase (u-PA) receptors at the cell surface (Vassalli *et al.*, 1985; Plow *et al.*, 1986) and hormones and growth factors of the extracellular milieu (Laiho & Keski-Oja, 1989). Epidermal growth factor (EGF) stimulates u-PA production (Grimaldi *et al.*, 1986; Niedbala & Sartorelli, 1989) whereas changes in the PA system induced by transforming growth factor-β (TGF-β) differ depending on the cell type studied. TGF-β enhances secretion of plasminogen activator inhibitor 1 (PAI-1) and u-PA of the human adenocarcinoma cell line A549 (Keski-Oja *et al.*, 1988) or decreases secretion of u-PA and tissue-type PA (t-PA) of embryonic fibroblasts (Lund *et al.*, 1987). In addition, TGF-β has been reported to have regulatory functions which counteract or modulate EGF responses (Keski-Oja *et al.*, 1987; Sporn *et al.*, 1987). Since these growth factors are potentially able to control the PA system with regard to its possible function in invasive growth of tumour cells we investigated the regulation of u-PA, t-PA and PAI secretion by EGF, TGF-α and TGF-β in malignant human lung carcinomas. Human lung tumours can be divided into non small cell lung carcinomas (NSCLC) and small cell lung carcinomas (SCLC) by their different clinical and physiological characteristics. The majority of NSCLC are squamous carcinomas, adenocarcinomas and large cell carcinomas. We used several human NSCLC cell lines which were of squamous cell origin (EPLC-65H, EPLC-272H, U-7152) and of large cell origin (LCLC-103H, LCLC-97TM1, U-1810). Some of these cell lines have previously been shown to produce and secrete components of the PA system, activators as well as inactivators, in different combinations and amounts (Heidtmann *et al.*, 1989). The cell lines have been described to express receptors for EGF (Haeder *et al.*, 1988) which also bind TGF-α. For stimulation experiments, the cells were cultivated in the presence of EGF, TGF-α, TGF-β or combinations of these factors as indicated in Figures 1 and 2. In addition we treated the cells with PMA which was shown to stimulate their synthesis of PA components (Heidtmann *et al.*, 1989). Secreted PA products were detected and resolved by their molecular mass using fibrin autography (Granelli-Piperno & Reich, 1978, Figure 1). Briefly, after separation of serum-free conditioned media in 8% SDS-gels, SDS was replaced by a non-ionic detergent, and proteins were allowed to diffuse into an indicator gel of 1% agarose, containing plasminogen and fibrin. Presence of PA-activity causes lysis zones in the detection gel. Overall PA activity was determined by a chromogenic assay (Figure 2).

Correspondence: R. Schwartz-Albiez, Institute of Immunology and Genetics, German Cancer Research Center, Im Neuenheimer Feld 280, D-6900 Heidelberg, Germany.
Received 10 June 1991; and in revised form 11 September 1991.

According to the secretion of u-PA, t-PA and the formation of PAI-complexes and alterations in their concentrations as a result of growth factor treatment, several principal patterns of PA constituents could be distinguished in the cell lines studied. All cell lines were able to produce u-PA. As judged from the intensity of the u-PA bands, growth factors influenced its activity only marginally or not at all. As an exception, cell line EPLC-65H which produced u-PA, t-PA and PAI at the detection limit showed marked u-PA secretion only after stimulation by growth factors or PMA. Significant effects of growth factors were observed in the secretion of t-PA and the formation of PAI-complexes. Cell lines EPLC-65H, EPLC-272H, U-1810 were able to produce t-PA and PAI. In these cell lines, t-PA secretion was either enhanced by EGF, TGF-α, PMA (EPLC-65H), or t-PA was already expressed endogenously with no apparent further increase under the influence of these agents (EPLC-272H and U-1810). Treatment with TGF-β alone or in combination with the other factors resulted in weaker expression of the t-PA band. On the other hand, TGF-β stimulated the formation of PAI-complexes as demonstrated for cell lines EPLC-272H and U-1810. The reduced amount of free t-PA under the influence of TGF-β may be due to two different mechanisms: either decreased secretion of t-PA as in cell line EPLC 65H, where the disappearance of t-PA is not accompanied by increased strength in PAI-complexes, or by complex formation of t-PA with additionally secreted PAI leading to stronger PAI-complex bands as in cell lines EPLC-272H and U-1810. The induction of PAI-complex bands in cell lines U-1752 and LCLC 103H under EGF, TGF-α and PMA, which was even more enhanced in the presence of TGF-β, indicates an increased turnover of PA, even though no t-PA bands appear, and u-PA bands are apparently not decreased in strength. Further, the total PA activity (Figure 2) is not decreased either but, in cell line LCLC-103H, appears to be increased in the presence of TGF-β instead. Thus it is conceivable, that in these cell lines under TGF-β, PA secretion is induced, but balanced by PAI. The induction of PA may either affect t-PA, which is then completely scavenged by PAI, or u-PA, which is then balanced to different degrees by concomitantly increased PAI. The cell lines differed in their PAI-complexes with regard to the band position and intensity in zymography after treatment with the respective factors. The microheterogeneity of upper and lower PAI-complex bands after TGF-β treatment, as observed with cell line EPLC-272H, may be caused by a different glycosylation of PAI. Although the nature of the PAI responsible for complexing could not be defined in these experiments, we presume that the cell lines predominantly produce PAI-1 since EPLC-65H and LCLC-103H were earlier found to synthesise PAI-1, and LCLC-103H additionally PAI-2 in small amounts (Heidtmann *et al.*, 1989).

The quantitative assessment of total PA activity (Figure 2) did not in all cases parallel the impressions gained from the fibrin autographies. It must be kept in mind that fibrin autography is, of course, a semiquantitative device. Thus, the over 3-fold increase in total PA activity in U-1752 under

**Figure 1** Fibrin autography of culture supernatants from human lung cancer cell lines. Zymographic detection of u-PA, t-PA and PAI-complexes was carried out as a modification of the method described by Granelli-Piperno and Reich, 1978. Samples were separated on slabs of 10% SDS-PAGE which were then placed on indicator gels containing 0.22 g agarose (agar noble, Difco, USA), 10 mg fibrinogen, 0.6 U thrombin and 0.8 mg plasminogen (all purchased from Behring Werke, Germany) in a total volume of 22 ml PBS. Standards consisted of u-PA (50 and 30 kDA) and t-PA (60 kDA) (Berhring Werke). The t-PA standard contained a trace contamination of u-PA. Sample sizes were adjusted to give optimal resolution, semiquantitative comparisons are thus only possible for samples of identical cell lines. Fibrin autography was incubated for 3 h. Prior to zymography cells were cultivated in serum-free RPMI 1640 containing 1% BSA for 2 days untreated (1) or treated with EGF (2), EGF/TGF-$\beta$ (3), TGF-$\alpha$ (4), TGF-$\alpha$/TGF-$\beta$ (5), PMA (6), PMA/TGF-$\beta$ (7) and TGF-$\beta$ (8). Growth factors and PMA were applied in concentrations assessed to be optimal in dose/response experiments using the chromogenic assay as described in Figure 2: PMA ($5 \times 10-9$ M), EGF and TGF-$\alpha$ (25 ng ml$^{-1}$), TGF-$\beta$ (10 ng ml$^{-1}$). PMA and EGF (purified from submaxillary gland of the mouse) were purchased from Sigma (USA); synthetic TGF-$\alpha$ from Bachem, USA; human platelet TGF-$\beta$1 from R + D Systemic Inc., USA. Growth factor/PMA treatment did not affect viability of the cells as determined by trypan blue exclusion. Supernatants were separated from cells by centrifugation at 400 *g*. In order to free the supernatants from cellular debris, culture media were centrifuged at 10,000 *g* for 20 min and the supernatants thereof were further used both for zymography and chromogenic assay.

**Figure 2** Plasminogen activator activity in culture supernatants of human lung cancer cell lines. PA activity was determined in supernatants of the cell lines under the influence of growth factors and PMA. Treatment was as described in Figure 1. Untreated cell cultures (1), treatment with EGF (2), EGF/TGF-$\beta$ (3), TGF-$\alpha$ (4), TGF-$\alpha$/TGF-$\beta$ (5), PMA (6), PMA/TGF-$\beta$ (7), TGF-$\beta$ (8). The chromogenic assay was a modification of the assay supplied by Kabi, Sweden (COA-SET): 2–100 $\mu$l of culture supernatant were brought to 100 $\mu$l with 0.05 M Tris-buffer containing 0.01% (v/v) Tween 20, pH 8.3 in microtiter plates, 0.15 ml of a mixture containing plasminogen (Behring Werke), substrate S-2251 (Kabi), and fibrinogen split products (Behring Werke) were added. Mock treatment with PMA or growth factors had no effect on the assay. For control, parallel assays were performed without plasminogen. Absorbance at 410 nm was measured after 75 min at 37°C in an automatic plate reader (Titertek Flow, Germany) and extrapolated to arbitrary units. Columns represent the mean values of triplicate measurements, bars represent SD, Representative values are shown out of a series of three independent experiments.

PMA (lane 6) is not directly evident from the respective u-PA band in Figure 1. In cell line EPLC-103H, the addition of TGF-β together with EGF, TGF-α or PMA led to increased total PA activity in spite of stronger inhibitor bands, indicating that a complementary increase in PA, either u-PA or t-PA, must have taken place. Thus, the action of TGF-β appears to involve the whole system and not only the inhibitory branch. We suggest that growth factors EGF, TGF-α and TGF-β modulate secretion of t-PA and PAI in these cell lines which are able to produce both PA's and PAI. Cell lines U-1752 and LCLC-103H are characterised by a deficiency in the production of t-PA. No significant effects of growth factors could be observed with these cell lines. In case of cell line LCLC-103H it seemed as if TGF-β even enhanced PA activity. These results support our notion that growth factors EGF, TGF-α and TGF-β primarily affect regulation of t-PA and PAI in a combined fashion. The mechanism underlying this regulation needs to be elucidated. LCLC-97TM1 cells, deficient in the production of PAI, showed no modulation of u-PA and t-PA secretion by the growth factors.

The patterns of the PA system and its regulation could not be correlated to the histological typing or grading of the original tumours which were squamous cell and large cell carcinomas of various degree of differentiation (Bepler *et al.*, 1988; Bergh *et al.*, 1981; Bergh *et al.*, 1985). In a previous study we had characterised a panel of NSCLC cell lines, comprising five of the cell lines used here, for their *in vitro* differentiation capacity and had found that PMA enhances the expression of several cellular differentiation markers (Salge *et al.*, 1990). EGF does not elicit all the responses seen under PMA, however, similar patterns of response of the PA system in these cell lines suggest that the induction of the PA system in NSCLC concurs with states of enhanced cellular differentiation.

In summary we presume that the expression of a complex pattern of PA and PAI, modified by EGF/TGF-α and TGF-β, may represent a general feature of NSCLC. Thus, the cells have an intricate and finely tuned set of tools for controlled proteolysis in their extracellular environment which includes mechanisms like fibrinolysis, tissue remodeling and facilitation of cell migration. Impairments may occur where proteolytic activity prevails over inhibitory activity. This would entail disturbance of the inhibitory balance leading to uncontrolled proteolysis. We cannot decide at the moment whether the dysregulations shown here for the cell lines represent true features of NSCLC or are phenomena acquired in cell culture. *In vivo* investigations of fresh tumour tissue samples may be complicated by the presence of a variety of non-tumour cells producing proteinases, inhibitors and growth factors. Therefore, cell culture experiments concerning the regulation of the PA system appear to be indispensable.

This work was in part supported by the SFB 215 of the German Research Community. We thank Dr Jonas Bergh, University of Uppsala, Sweden, for providing us with the cell lines U-1752 and U-1810. The excellent technical assistance of Karin Beisenherz is gratefully acknowledged.

### References

BEPLER, G., KOEHLER, A., KIEFER, P. & 5 others (1988). Characterization of the state of differentiation of six newly established human non-small-cell lung cancer cell lines. *Differentiation*, **37**, 158.

BERGH, J., NILSSON, K., ZECH, L. & GIOVANELLA, B. (1981). Establishment and characterization of a continuous lung squamous cell carcinoma cell line (U-1752). *Anticancer Res.*, **1**, 317.

BERGH, J., NILSSON, K., EKMAN, R. & GIOVANELLA, B. (1985). Establishment and characterization of cell lines from human small cell and large cell carcinomas of the lung. *Acta. Path. Microbiol. Scand. Sect. A*, **93**, 133.

CAJOT, J.F., SCHLEUNIG, W.D., MEDCALF, R.L. & 4 others (1989). Mouse L cells expressing human prourokinase-type plasimogen activator: effects on extracelluiar matrix degradation and invasion. *J. Cell Biol.*, **109**, 915.

GRANELLI-PIPERNO, A. & REICH, E. (1978). A study of proteases and protease-inhibitor complexes in biological fluids. *J. Exp. Med.*, **148**, 223.

GRIMALDI, G., DI FIORE, P., LOCATELLI, E.K., FALCO, J. & BLASI, F. (1986). Modulation of urokinase plasminogen activator gene expression during the transition from quiescent to proliferative state in normal mouse cells. *EMBO J.*, **5**, 855.

HAEDER, M., ROTSCH, M., BELPER, G. & 4 others (1988). Epidermal growth factor receptor expression in human lung cancer cell lines. *Cancer Res.*, **48**, 1132.

HEIDTMANN, H.-H., HOFMANN, M., JACOB, E., ERBIL, C., HAVEMANN, K. & SCHWARTZ-ALBIEZ, R. (1989). Synthesis and secretion of plasminogen activators and plasminogen activator inhibitors in cell lines of different groups of human lung tumors. *Cancer Res.*, **49**, 6960.

KESKI-OJA, J., LEOF, E.B., LYONS, R.M., COFFEY, R.J. & MOSES, H.L. (1987). Transforming growth factors and control of neoplastic cell growth. *J. Cell. Biochem.*, **33**, 95.

KESKI-OJA, J., BLASI, F., LEOF, E.B. & MOSES, H.L. (1988). Regulation of the synthesis and activity of urokinase plasminogen activator in A549 human lung carcinoma cells by transforming growth factor-β. *J. Cell Biol.*, **106**, 451.

LAIHO, M. & KESKI-OJA, J. (1989). Growth factors in the regulation of pericellular proteolysis: a review. *Cancer Res.*, **49**, 2533.

LUND, L.R., RICCIO, A., ANDREASEN, P.A. & 5 others (1987). Transforming growth factor-β is a strong and fast acting positive regulator of the level of type-1 plasminogen activator inhibitor mRNA in WI-38 human lung fibroblasts. *EMBO J.*, **6**, 1281.

NIEDBALA, M.J. & SARTORELLI, A.C. (1989). Regulation by epidermal growth factor of human squamous cell carcinoma plasminogen activator-mediated proteolysis of extracellular matrix. *Cancer Res.*, **49**, 3302.

PLOW, E.F., FREANEY, D.E., PLESCIA, J. & MILES, L.A. (1986). The plasminogen system and cell surfaces: evidence for plasminogen and urokinase receptors on the small cell type. *J. Cell Biol.*, **103**, 2411.

SALGE, U., KILIAN, P., NEUMANN, K., ELSAESSER, H.P., HAVEMANN, K. & HEIDTMANN, H.H. (1990). Differentiation capacity of human non-small-cell lung cancer cell lines after exposure to phorbol ester. *Int. J. Cancer*, **45**, 1143.

SPORN, M.B., ROBERTS, A.B., WAKEFIELD, L.M. & DE CROMBRUGGHE, B. (1987). Some recent advances in the chemistry and biology of transforming growth factor-β. *J. Cell Biol.*, **105**, 1039.

TANAKA, K., KOHGA, S., KINJO, M. & KODAMA, Y. (1977). Tumor metastasis and thrombosis, with special reference to thromboplastic and fibrinolytic activities of tumor cells. *Gann*, **20**, 97.

VASSALLI, J.-D., BACCINO, D. & BELIN, D. (1985). A cellular binding site for the Mr 55,000 form of the human plasminogen activator, urokinase. *J. Cell Biol.*, **100**, 86.


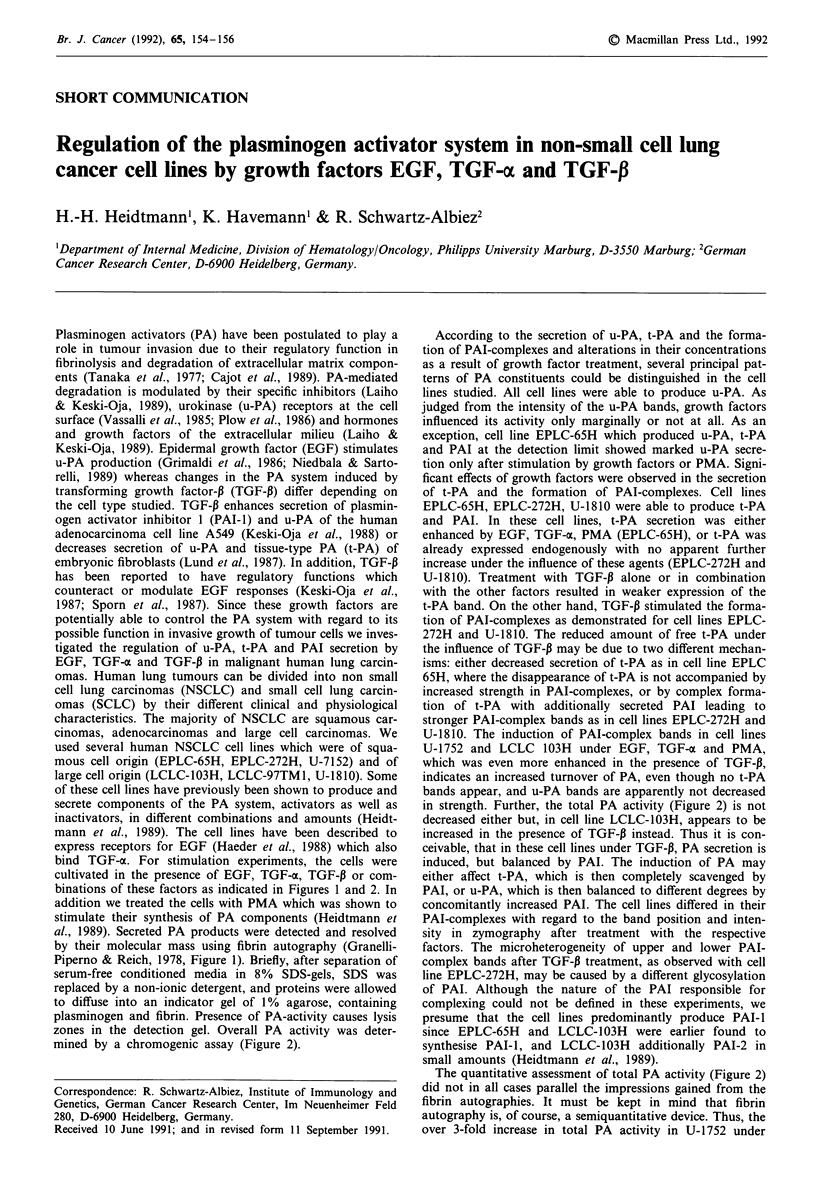

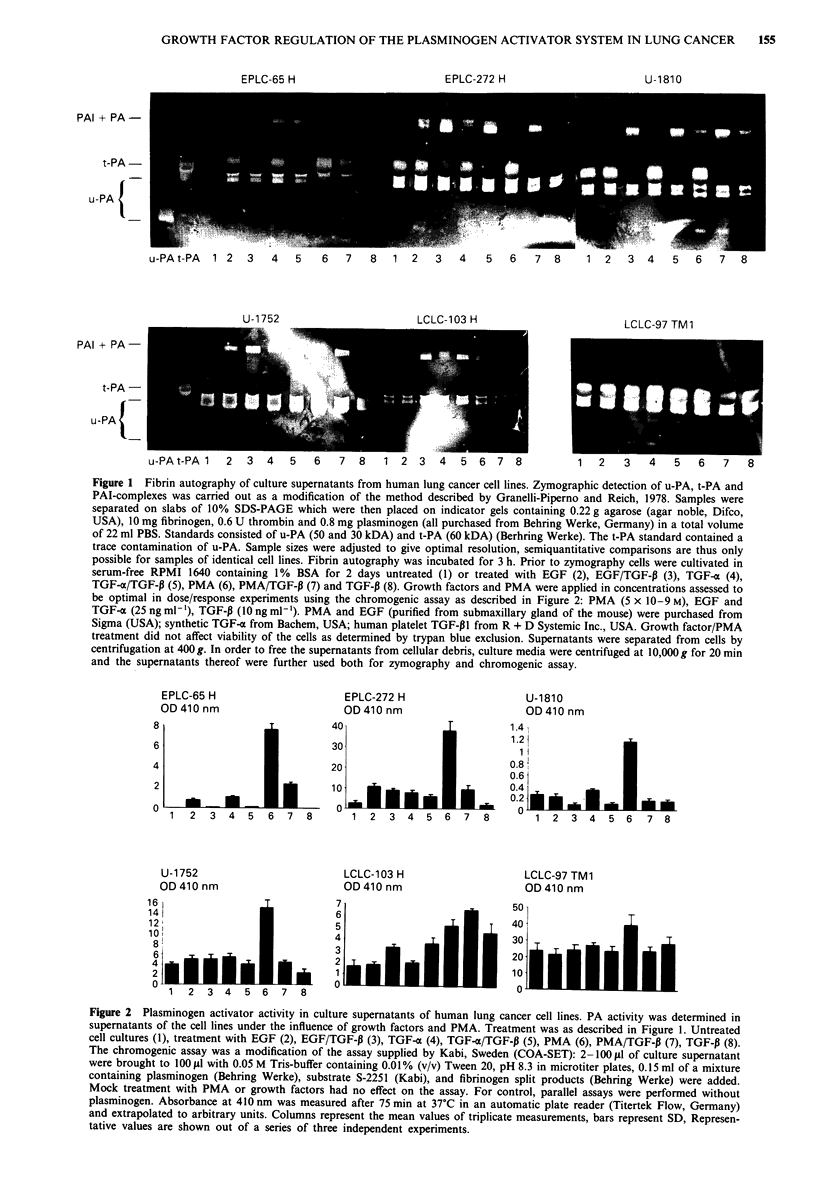

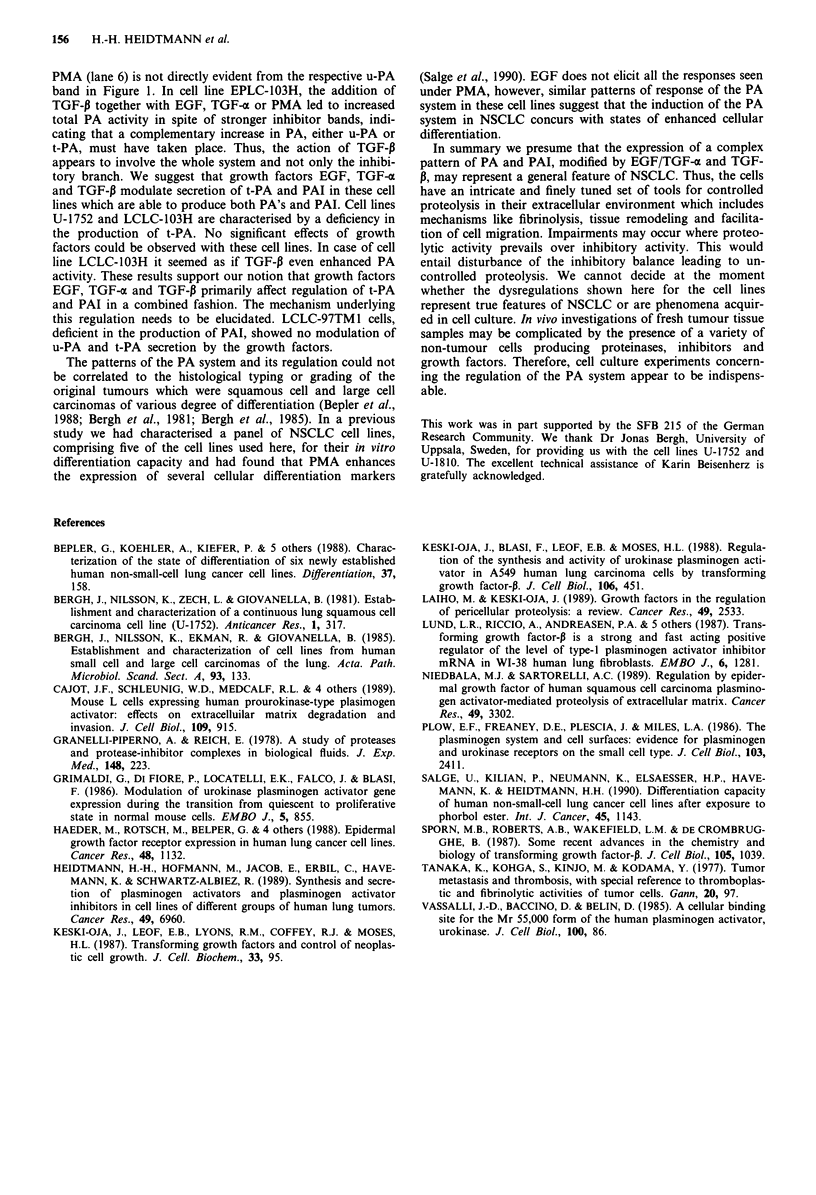


## References

[OCR_00288] Bepler G., Koehler A., Kiefer P., Havemann K., Beisenherz K., Jaques G., Gropp C., Haeder M. (1988). Characterization of the state of differentiation of six newly established human non-small-cell lung cancer cell lines.. Differentiation.

[OCR_00299] Bergh J., Nilsson K., Ekman R., Giovanella B. (1985). Establishment and characterization of cell lines from human small cell and large cell carcinomas of the lung.. Acta Pathol Microbiol Immunol Scand A.

[OCR_00294] Bergh J., Nilsson K., Zech L., Giovanella B. (1981). Establishment and characterization of a continuous lung squamous cell carcinoma cell line (U-1752).. Anticancer Res.

[OCR_00305] Cajot J. F., Schleuning W. D., Medcalf R. L., Bamat J., Testuz J., Liebermann L., Sordat B. (1989). Mouse L cells expressing human prourokinase-type plasminogen activator: effects on extracellular matrix degradation and invasion.. J Cell Biol.

[OCR_00311] Granelli-Piperno A., Reich E. (1978). A study of proteases and protease-inhibitor complexes in biological fluids.. J Exp Med.

[OCR_00316] Grimaldi G., Di Fiore P., Locatelli E. K., Falco J., Blasi F. (1986). Modulation of urokinase plasminogen activator gene expression during the transition from quiescent to proliferative state in normal mouse cells.. EMBO J.

[OCR_00322] Haeder M., Rotsch M., Bepler G., Hennig C., Havemann K., Heimann B., Moelling K. (1988). Epidermal growth factor receptor expression in human lung cancer cell lines.. Cancer Res.

[OCR_00329] Heidtmann H. H., Hofmann M., Jacob E., Erbil C., Havemann K., Schwartz-Albiez R. (1989). Synthesis and secretion of plasminogen activators and plasminogen activator inhibitors in cell lines of different groups of human lung tumors.. Cancer Res.

[OCR_00339] Keski-Oja J., Blasi F., Leof E. B., Moses H. L. (1988). Regulation of the synthesis and activity of urokinase plasminogen activator in A549 human lung carcinoma cells by transforming growth factor-beta.. J Cell Biol.

[OCR_00334] Keski-Oja J., Leof E. B., Lyons R. M., Coffey R. J., Moses H. L. (1987). Transforming growth factors and control of neoplastic cell growth.. J Cell Biochem.

[OCR_00345] Laiho M., Keski-Oja J. (1989). Growth factors in the regulation of pericellular proteolysis: a review.. Cancer Res.

[OCR_00349] Lund L. R., Riccio A., Andreasen P. A., Nielsen L. S., Kristensen P., Laiho M., Saksela O., Blasi F., Danø K. (1987). Transforming growth factor-beta is a strong and fast acting positive regulator of the level of type-1 plasminogen activator inhibitor mRNA in WI-38 human lung fibroblasts.. EMBO J.

[OCR_00355] Niedbala M. J., Sartorelli A. C. (1989). Regulation by epidermal growth factor of human squamous cell carcinoma plasminogen activator-mediated proteolysis of extracellular matrix.. Cancer Res.

[OCR_00361] Plow E. F., Freaney D. E., Plescia J., Miles L. A. (1986). The plasminogen system and cell surfaces: evidence for plasminogen and urokinase receptors on the same cell type.. J Cell Biol.

[OCR_00369] Salge U., Kilian P., Neumann K., Elsässer H. P., Havemann K., Heidtmann H. H. (1990). Differentiation capacity of human non-small-cell lung cancer cell lines after exposure to phorbol ester.. Int J Cancer.

[OCR_00375] Sporn M. B., Roberts A. B., Wakefield L. M., de Crombrugghe B. (1987). Some recent advances in the chemistry and biology of transforming growth factor-beta.. J Cell Biol.

[OCR_00382] Vassalli J. D., Baccino D., Belin D. (1985). A cellular binding site for the Mr 55,000 form of the human plasminogen activator, urokinase.. J Cell Biol.

